# Ox-LDL Induces ER Stress and Promotes the adipokines Secretion in 3T3-L1 Adipocytes 

**DOI:** 10.1371/journal.pone.0081379

**Published:** 2013-11-22

**Authors:** Yaqin Chen, Mingjie Chen, Zhihong Wu, Shuiping Zhao

**Affiliations:** Department of Cardiology, The Second Xiangya Hospital of Central South University, Central South University, Changsha, Hunan, P.R China; Universidade Federal do Rio de Janeiro, Brazil

## Abstract

Adipocytes behave as a rich source of adipokines, which may be the link between obesity and its complications. Endoplasmic reticulum (ER) stress in adipocytes can modulate adipokines secretion. The aim of this study is to evaluate the effect of oxidized low density lipoprotein（ox-LDL）treatment on ER stress and adipokines secretion in differentiated adipocytes. 3T3-L1 pre-adipocytes were cultured and differentiated into mature adipocytes in vitro. Differentiated adipocytes were incubated with various concentrations of ox-LDL (0-100 µg/ml) for 48 hours; 50µg/ml ox-LDL for various times (0-48 hours) with or without tauroursodeoxycholic acid (TUDCA) (0-400µM) pre-treatment. The protein expressions of ER stress markers, glucose regulated protein 78(GRP78) and CCAAT/enhancer binding protein [C/EBP] homologous protein (CHOP) in adipocytes were detected by Western blot. The mRNA expressions of visfatin and resistin were measured by real-time PCR and the protein release of visfatin and resistin in supernatant were determined by ELISA. Treatment with ox-LDL could increase the cholesterol concentration in adipocytes. Ox-LDL induced the expressions of GRP78 and CHOP protein in adipocytes and promoted visfatin and resistin secretion in culture medium in dose and time-dependent manner. TUDCA could attenuate the effect of ox-LDL on GRP78 and CHOP expressions and reduce visfatin and resistin at mRNA and protein level in dose-dependent manner. In conclusion, ox-LDL promoted the expression and secretion of visfatin and resistin through its activation of ER stress, which may be related to the increase of cholesterol load in adipocytes.

## Introduction

In addition to storage of energy as a passive reservoir, adipose tissue is also considered as an important endocrine organ that produces and secretes a variety of bioactive molecules called adipokines, such as tumor necrosis factor-α (TNF-α), monocyte chemoattractant protein 1 (MCP-1),adiponectin and plasminogen activator inhibitor type 1 (PAI-1)[1–4]. Visfatin and resistin represents novel adipokines of the visceral adipose tissue [5,6]. Obesity is attributed to excessive adipose deposition, characterized by hypertrophy and hyperplasia of adipocytes. Secretory profile of adipocytes in obesity is shifted towards the proinflammatory spectrum. The abnormalities of the expression and secretion of adipokines may be the link between obesity and its complications. 

Newly synthesized secretory and membrane-associated proteins are correctly folded and assembled by chaperones in the endoplasmic reticulum (ER) [7]. Failure of the ER's adaptive capacity results in activation of the ER stress, also known as unfolded protein response (UPR).Recent studies have reported that ER stress is increased in liver and adipose tissue of obese mice [8,9] and obese human subjects [10]. Induction of ER stress in fat tissues modifies adipokines secretion and induces inflammation [11]. So it could be presumed that inhibition of ER stress may be an effective approach to reduce the risk of obesity and its complications. However, the triggers that induce ER stress in obesity have not been fully elucidated.

Circulating oxidized low-density lipoprotein (ox-LDL) is significantly correlated with most of the proatherogenic risk factors including obesity, dyslipidemia and metabolic syndrome [12]. Ox-LDL triggers various biological responses potentially involved in atherosclerosis-related disease by triggering lipid storage, local inflammation and toxic events.

 [13]. Recent studies indicated that ox-LDL could trigger ER stress in endothelial cells and macrophages, which is dependent on cholesterol trafficking to the endoplasmic reticulum [14–16]. However it is rarely reported about the effect of ox-LDL on ER stress and subsequent adipokines secretion in adipocytes. In the present study, we showed that ox-LDL induces ER stress in adipocytes which maybe partly due to intracellular cholesterol overload. Furthermore, we found that alleviation of ER stress using chemical chaperones modified the inflammatory adipokines secretion.

## Materials and Methods

### 2.1. Cell culture and treatment

3T3-L1 preadipocytes purchased from The American Type Culture Collection (ATCC) were maintained in DMEM-F12 (GIBCO) supplemented with 10% FBS (basal medium). The cells were then cultured in 5% CO_2_ at 37 °C. For the induction of adipocytes differentiation, cells were 1) precultured in basal medium for 2 days and grown to confluence, 2) treated with differentiation medium containing 10 μg/ml insulin, 0.25 μM dexamethasone, and 500 μM IBMX (IDI medium) for 2 days, and 3) incubated in basal medium supplemented with insulin alone for 2 days. The cells were further incubated in basal medium for an additional 2 days .At that time, greater than 90% of cells had accumulated multiple lipid droplets and adipocytes differentiation was achieved, which was identified by Oil red O staining method. For the experiment, cells were plated in 6-well plates at a density of 1.5×10^6^ cells/ml.

When appropriate, the differentiated 3T3-L1 cells were harvested and washed three times in warm PBS. Cells were incubation with DMEM+0.2% BSA at 37 °C for 12 hrs and then were FC-loaded by incubation with full medium containing 10 μg/ml of the ACAT inhibitor 58035 (Sandoz, Inc) （10µg/ml) plus ox-LDL (0 to 100µg/ml) for 48 hrs or 58035 10 μg/ml plus ox-LDL 50 µg/ml for 0h to 48 hrs. LDL (d, 1.020–1.063 g/ ml^−1^) from fresh human plasma was isolated by preparative ultracentrifugation and then oxidized. To further verify whether the effect of ox-LDL on adipocytes is associated with ER stress activation, the adipocytes were pretreated for 12 hours with various doses of TUDCA(580549, Calbiochem, Gibbstown, NJ)) ( 0- 400µM) ,a chemical chaperone known to ameliorate ER stress [15], and then stimulated with 50 μg/ml of ox-LDL plus ACAT inhibitor 58035 for 48 hours. At the end of the study, the supernatants and monolayer cells were harvested for next experiments.

### 2.2. Cellular cholesterol quantification

The total and free cholesterol were analyzed using the Cholesterol/Cholesteryl Ester Quantitation Kit (Biovision). In brief, the cells were washed twice with PBS; lipids were extracted by the addition of 1 ml chloroform/methanol (2:1) to the cell pellet. After sonification, the sample was centrifuged and the lipid phase was collected. Then, 0.5 ml of 0.9% NaCl was added to the liquid and the lipid layer in the bottom of tube was carefully collected. The sample was then dried in vacum and samples were dissolved in 95% ethanol. Cholesterol ester was converted to free cholesterol by cholesterol ester hydrolase for determination of total cholesterol. Cholesterol oxidase was employed to generate H_2_O_2_ from free cholesterol, and peroxidase was used to catalyze the reaction of H_2_O_2_ with p-hydroxyphenylacetic acid to yield a stable fluorescent product. The concentration of total and free cholesterol per well was analyzed using a standard curve and normalized by measuring the concentration of total cell protein using the Lowry protein assay. Cholesteryl ester can be determined by subtracting the value of free cholesterol from the total (cholesterol plus cholesteryl esters). 

### 2.3. Western blotting

Cell lysates were prepared with RIPA lysis solution (Beyotime Institute of Biotechnology, China). Protein concentration was determined using the bicinchoninic acid (BCA) protein assay kit (Pierce).Equivalent amounts of protein were denatured and subjected to 15% sodium dodecyl sulfate-polyacrylamide gel electrophoresis (SDS-PAGE). After gel separation, the proteins were transferred to a PVDF membrane. The primary antibodies are diluted as the manufacturer's recommendations: mouse anti-GRP78, mouse nti-GADD153/C/EBP homologous protein (CHOP) and mouse anti-β actin antibody (Sigma). GRP78 and CHOP antibody were acquired from Santa Cruz Biotechnology (Santa Cruz, CA).The membrane were incubated for 1 h with either anti-mouse or anti-rabbit horseradish peroxidase IgG secondary antibodies (Sigma).Chemiluminescence detection using Western Lightning Chemiluminescence Reagent plus was performed. Membranes were exposed to imaging film (Kodak Bioflex Econo Scientific) and developed using a Kodak X-OMAT 1000A. The immunoreactive band was visualized by using the ECL detection reagent (Applygen Tech Inc, China). The densities were measured using a scanning densitometer coupled to scanning software (ImageQuant; Molecular Dynamics, Amersham, Little Chalfont, UK). The expression of GRP78 and CHOP was evaluated and compared with the expression of β-actin.

### 2.4. RNA isolation and real-time PCR

The mRNA expressions of visfatin and resistin were evaluated by the method of real-time PCR. Total RNA was extracted from adipocytes using Trizol reagent

(Invitrogen) according to manufacturer’s instructions. RNA was reverse transcribed using SuperScript III First-Strand Synthesis Supermix (Invitrogen). The cDNA samples were amplified in duplicate in 96-microtiter plates (Applied Biosystems).

Each PCR reaction (20 μl of total volume) contained: 10 μl of SYBR Green PCR

Master Mix (Applied Biosystems), 5 pmols of each primer, 1 μg of cDNA. The PCR

primers were the following: 1) visfatin: 5'- ATT TGG CCA TCC CCC TTC TG-3' and

5'- GGG TGA CAC GCA AAT CAA CTC-3'; 2) resistin: 5'- CTT CAA CTC CCT GTT TCC AAA TGC-3' and 5'- CCA CAG GAG CAG CTC AAG AC-3'. Real-time PCR reactions were carried out in an ABI PRISM 7,500 real-time PCR apparatus. The thermal profile settings were 95°C for 2 min, then 40 cycles at 95°C for 10 s, 60°C for 30 s and 70°C for 45 s. The relative mRNA expression levels were normalized to expression of 28S rRNA.

### 2.5. ELISA

Mouse visfatin (MBL International, Woburn, MA) and resistin (R&D Systems, Inc) ELISA kits were used to assay secreted visfatin and resistin from cultured 3T3-L1 cells. Assays were performed as per the manufacturer's protocol. All samples were measured in triplicate. Intra-assay precision variability was < 4% and 5.8% respectively.

### 2.6. Statistical analysis

All data are presented as means ± SD of triplicate experiments. Comparisons among groups were performed by one-way ANOVA analysis with Bonferroni’s test for post hoc analysis. Differences were considered significant at a value of P<0.05 for all tests. 

## Results

### 3.1. Effect of ox-LDL on expression and secretion of visfatin and resistin in 3T3-L1 adipocytes

**Figure 1:**
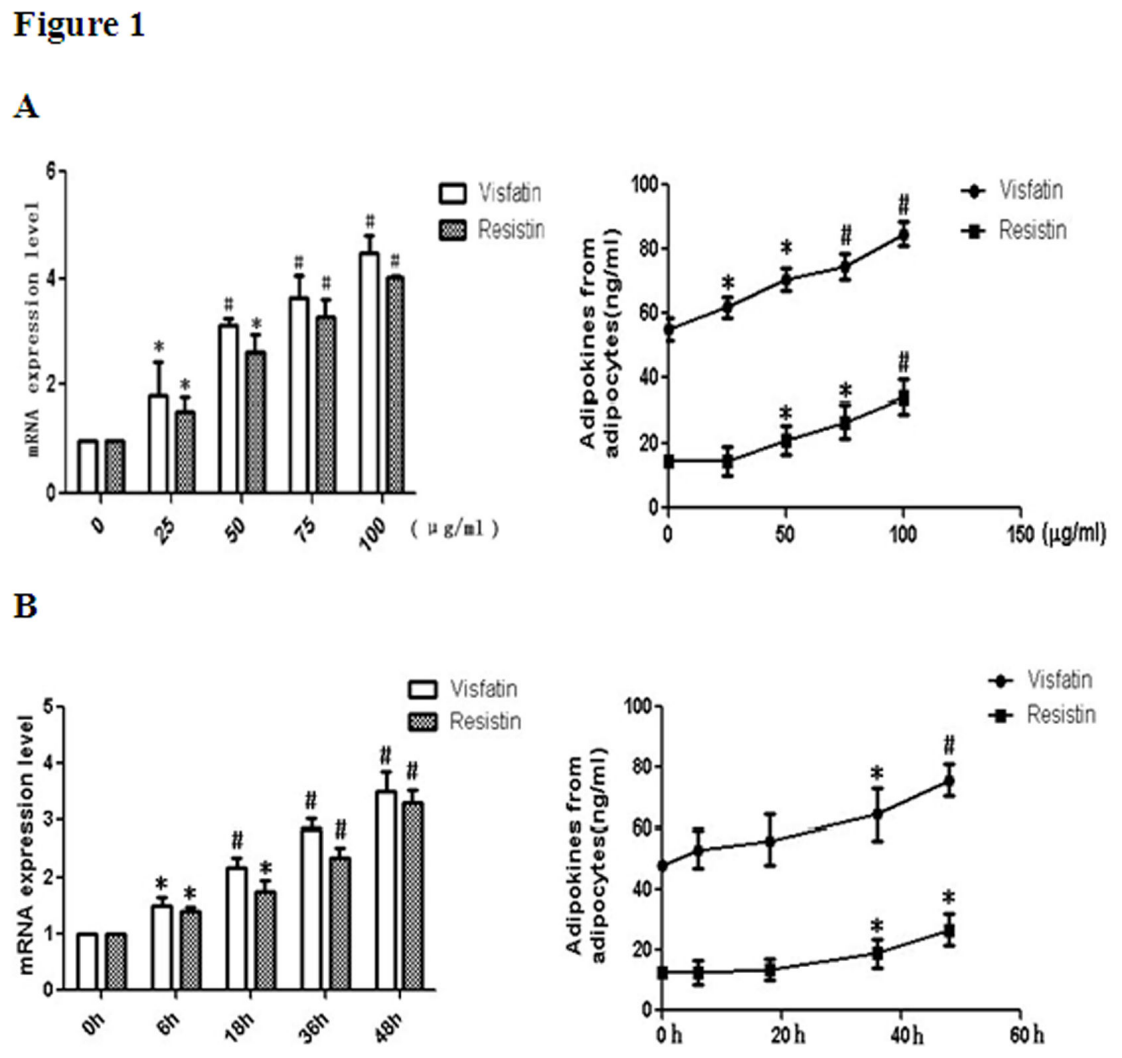
Ox-LDL loading induces the secretion of visfatin and resistin in adipocytes. Differentiated 3T3-L1 adipocytes were incubated in medium containing various concentration of ox-LDL (0, 25, 50， 75， 100µg/ml) and 10µg/ml Sandoz 58035 for 48hrs(A); Adipocytes were incubated with ox-LDL (50µg/ml) in a combination of sandoz58035(10µg/ml) for various times(B). At the end of the incubation, the mRNA expressions of visfatin and resistin were evaluated by real-time PCR. The culture media were collected, and the amounts of visfatin and resistin released to the media were analyzed by enzyme-linked immunosorbent assay. * P<0.05, as compared with control; # P<0.01, as compared with control. Data are presented as means ± SD (n = 3).

In the present study, we examined the adipokines levels of adipocytes after treatment with ox-LDL by measuring the mRNA expression and secretion of visfatin and resistin. Incubation of adipocytes with ox-LDL led to significant up-regulation of visfatin and resistin release in a dose-dependent manner with the increasing concentrations of ox-LDL (Figure 1A). At the same time, the mRNA expression of visfatin and resistin were also dose-dependently increased after ox-LDL intervention (Figure 1A).

**Figure 2:**
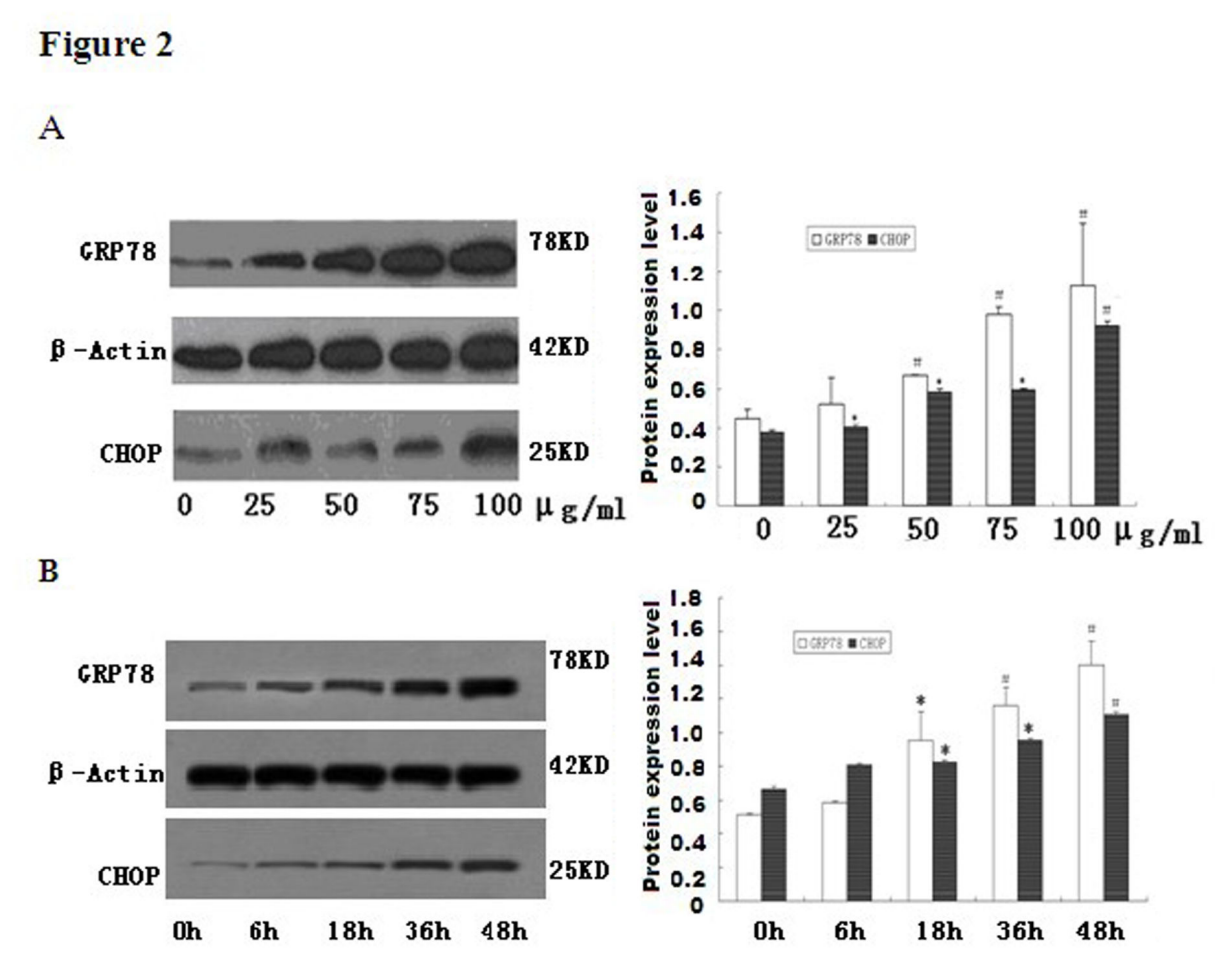
Free cholesterol induction of ER stress in adipocytes. Fully differentiated 3T3-L1 cells were incubated in the medium containing various concentration of ox-LDL (0-100µg/ml) with Sandoz 58035 for 48 hrs (A). Lane 1: control; lane 2: ox-LDL at 25μg/ml; lane 3: ox-LDL at 50μg/ml; lane 4: ox-LDL at 75μg/ml; lane 5: ox-LDL at 100μg/ml; Adipocytes were incubated with ox-LDL (50µg/ml) in a combination of Sandoz58035 for indicated times (0-48hrs) (B). Lane 1: control; lane 2: ox-LDL for 6h; lane 3: ox-LDL for 18h; lane 4: ox-LDL for 36h; lane 5: ox-LDL for 48h. GRP78 and CHOP expression was assessed by Western Blot andβ-actin was used as the housekeeping gene for normalization. Date is expressed as mean ±S.D from at least three independent determinations. * P<0.05, as compared with control; # P<0.01, as compared with control.

We also investigated the time-dependent effect of ox-LDL on visfatin and resistin secretion from 3T3-L1 adipocytes. Results shown in Figure 2B indicated that the gene expression levels of visfatin and resistin were increased in a time-dependent manner with the increasing incubation time of ox-LDL. Visfatin and resistin concentration in adipocytes liquid supernatant were also up-regulated in a time-dependent manner (Figure 2B).

### 3.2. Ox-LDL induced ER stress in adipocytes

To determine whether cholesterol induces ER stress in 3T3-L1 adipocytes, we investigated the expression patterns of several molecular indicators of ER stress in these cells. Western blot analysis demonstrated marked accumulation of GRP78 and CHOP protein in cell lysates after ox-LDL incubation as compared with that in the control. Consistent with the increasing levels of adipokines by the stimulating effect of ox-LDL, the GRP78 and CHOP protein expression in cholesterol load adipocytes were also significantly enhanced in both a dose-dependent (Figure 2A) and time-dependent manner (Figure 2B). These results indicated that ox-LDL may cause ER stress in 3T3-L1 adipocytes.

### 3.3. Chemical Chaperone Treatment Protects cells from ER Stress and secretion dysfunction

**Figure 3:**
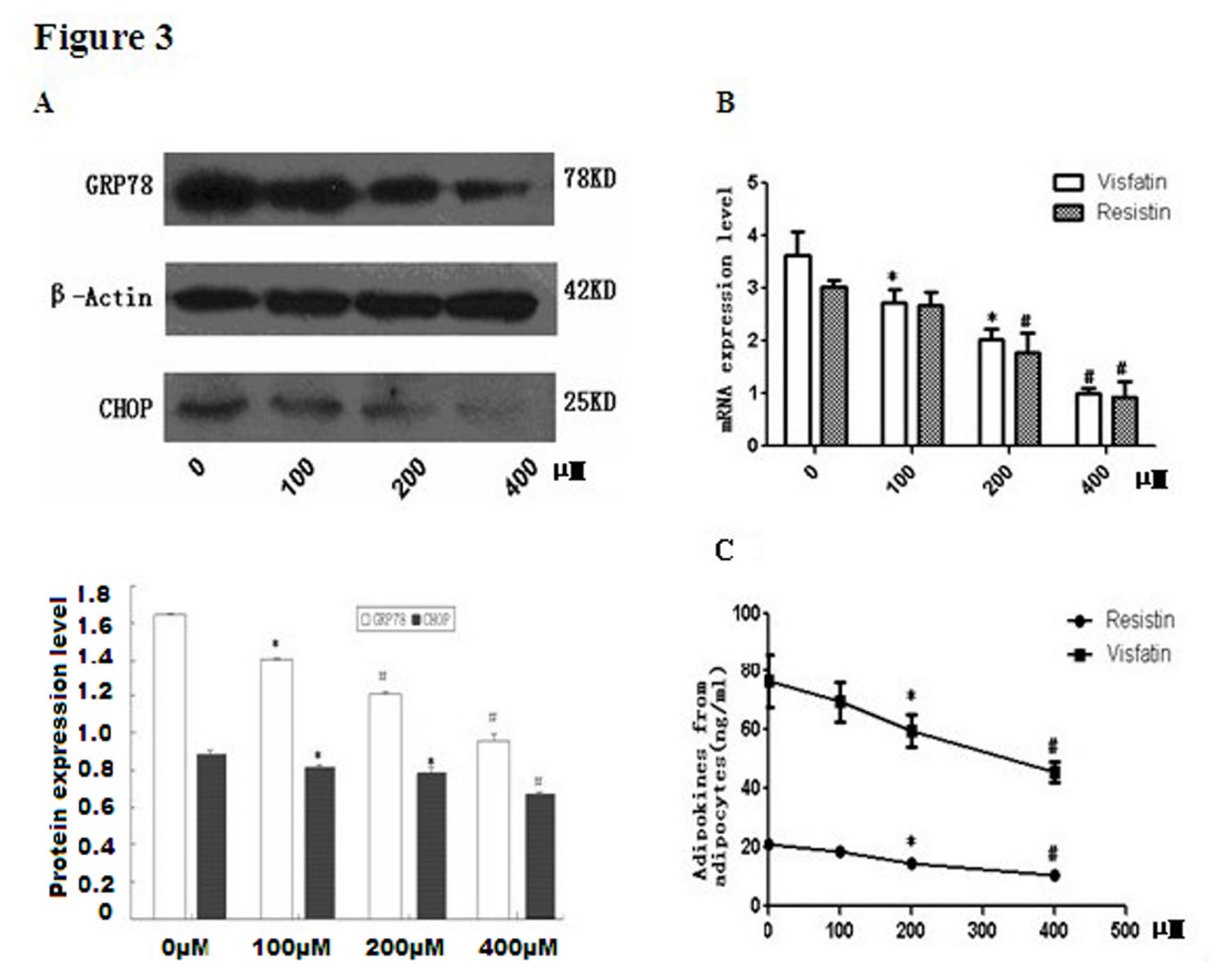
Chemical Chaperone Treatment Protects cells from secretion dysfunction. Differentiated 3T3-L1 adipocytes were incubated in the medium with ox-LDL(50µg/ml) ，Sandoz58035 and different concentration of TUDCA (0-400µM) for 48hrs. GRP78 and CHOP expression was determined by Western Blot and β-actin was used as the housekeeping gene for normalization(A). Lane 1: control; lane 2: TUDCA at 100µM; lane 3: TUDCA at 200µM; lane 4: TUDCA at 400µM. The mRNA expressions of resistin and visfatin were evaluated by real-time PCR (B).Resistin and visfatin release from 3T3-L1 adipocytes into supernatant medium were analyzed by ELISA (C). * P<0.05, as compared with control; # P<0.01, as compared with control.

TUDCA is a well-established chemical chaperone and ER stress inhibitor [15]. To further explore whether the stimulating effect of ox-LDL on adipokines secretion is attributed to the activation of ER stress, differentiated adipocytes were treated with TUDCA before exposure to ox-LDL. As compared with adipocytes treated with ox-LDL alone, GRP78 and CHOP protein expression level significantly decreased in TUDCA pre-treatment group as expected (Figure 3A)，indicating that ER-stress was effectively suppressed. Also, TUDCA treatment significantly protected the cells from FC induced inflammatory adipokines accumulation (Figure 3B). Consistently, the mRNA expression of visfatin and resistin induced by ox-LDL markedly alleviates in 3T3-L1 adipocytes by TUDCA pre-incubation in a dose dependent manner (Figure 3C).

### 3.4. Intracellular free cholesterol increased after ox-LDL intervention

**Figure 4:**
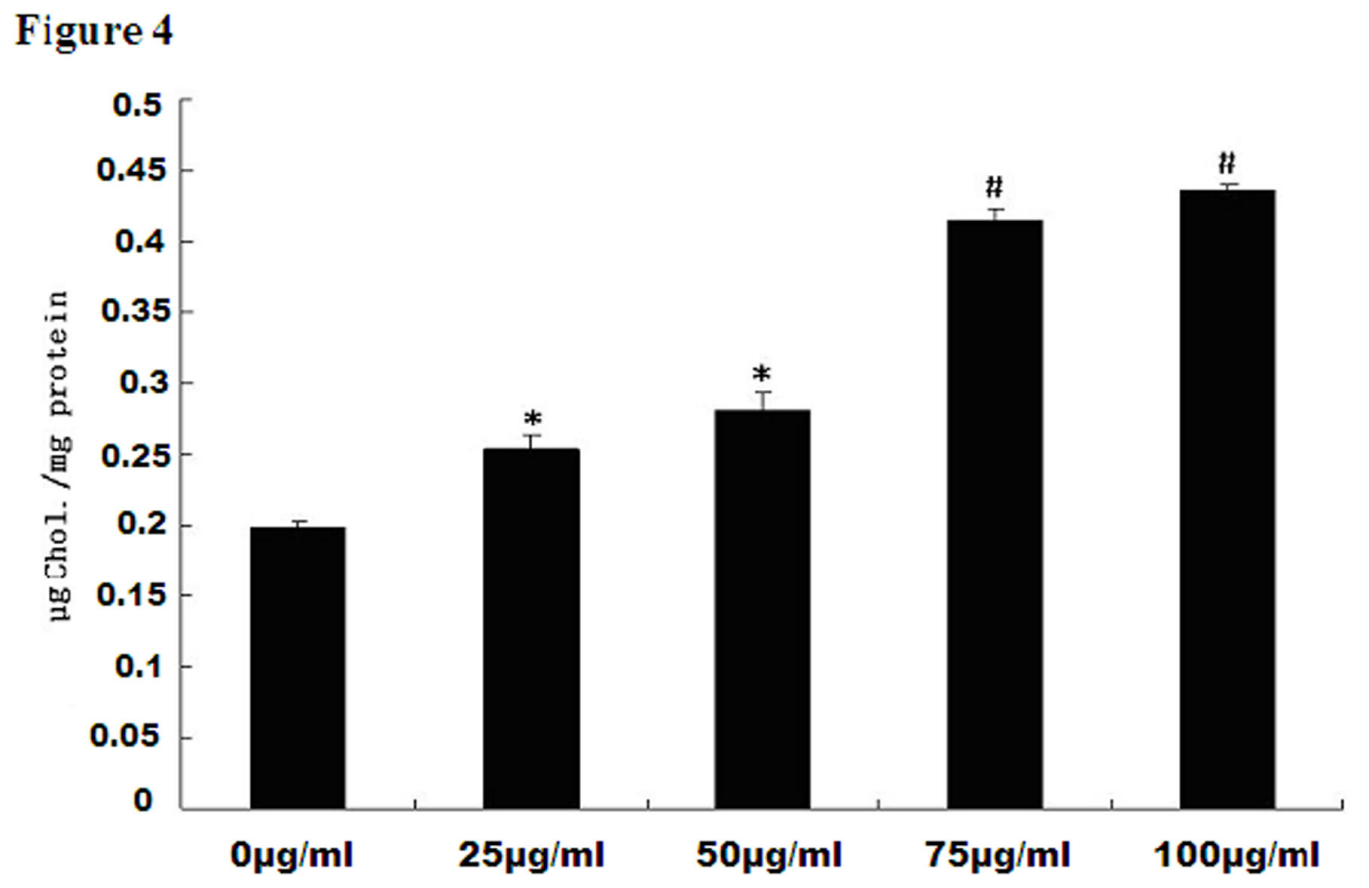
Intracellular free cholesterol concentration after ox-LDL intervention. Differentiated 3T3-L1 adipocytes were exposed to increasing concentrations (0, 25, 50， 75， 100µg/ml) of ox-LDL and 10µg/ml ACAT inhibitor Sandoz 58035. Total cell cholesterol level in adipocytes were increased in a dose-dependent manner with ox-LDL intervention. Dates are expressed as mean ±S.D from at least three independent determinations. * P<0.05, as compared with control; # P<0.01, as compared with control.

We examined endocytic uptake of FC using the differentiated 3T3-L1 cell line. Ox-LDL is a rich source of FC. ACAT inhibitor Sandoz 58035 was used to inhibit FC transforming into cholesterol ester in order to enhance cellular FC concentration. A series of experiments with increasing concentrations (0, 25, 50， 75， 100µg/ml) of ox-LDL plus the ACAT inhibitor 58035 was performed. Outcome parameters were compared with control. As shown in Figure 4, the total uptakes of ox-LDL in adipocytes were increased in a concentration-dependent manner. Compared with cholesterol concentration in adipocytes without ox-LDL treatment, ox-LDL at 25, 50， 75， 100 µg/ml significantly increased intracellular cholesterol concentration by 1.3, 1.4, 2.1, and 2.2-fold respectively (P<0.05). 

## Discussion

Recently many studies indicated that ER stress has emerged as a factor that is relevant to obesity-induced insulin resistance and diabetes [16].ER stress in obesity can disturb the secretory function of adipocytes [17]. Published evidence showed that ER stress significantly lowered the accumulation of adiponectin and leptin [18,19].Inflammatory cytokines such as IL-6 was strongly induced by ER stress activation [19]. Lefterova et al. claimed ER stress inducer tunicamycin and thapsigargin(TG) inhibited resistin transcription in murine adipocytes through up-regulation of CHOP [20]. In contrast, an interesting finding in this study was that ox-LDL-activating-ER stress induced increasing levels of visfatin and resistin expression in 3T3-L1 adipocytes. It is possible that different types of ER stress inducers can oppositely affect the expression of adipokines. Ox-LDL may also regulate adipokines release of adipocytes by some other signaling pathway beyond up-regulating CHOP expression. Previous studies also showed opposite regulation of leptin by different ER stress inducer [19,20]. We further demonstrated that chemical enhancement of ER function by TUDCA partially abolishes ER stress induction and restores visfatin and resistin release. All these data suggested that the accumulation of visfatin and resistin expression and secretion might be, at least partly, due to the stimulation of ER stress, which supported the concept that ER stress may be the proximal cause of inflammation in adipocytes. Therefore, our results provided evidence that ER stress may be a potential therapeutic target for the treatment for obesity related inflammation.

The triggers that induce ER stress in obesity remained unclear. Various intracellular and extracellular stimuli including glucose or nutrient deprivation, hypoxia, oxidative stress and increased synthesis of secretory proteins can trigger ER stress [21]. Ox-LDL has a wide range of biological properties including up-regulation of inflammatory genes, increased expression of adhesion molecules on endothelial cells, monocyte chemotaxis and destabilization of plaques. Previous studies reported that ox-LDL triggers ER stress in vascular cells and macrophages through activation of the ER stress sensors IRE1α, eIF2α and ATF6 and subsequently leads to cellular dysfunction1[22,23]. In our research, as the ox-LDL concentration increased，the protein express of GRP78 and CHOP protein—important ER stress biomarkers were markedly induced in adipocytes and the secretion function was affected. To some extent, these results may help explain the close link between obesity related hyperlipidemia and adipose tissue inflammation.

The mechanism by which ox-LDL triggered ER stress in adipocytes is complex and need to be further elucidated. It is well-known that cholesterol is not only considered as a structural component but also actively participates in the regulation of cell physiology [24]. In fact, excess free cholesterol (FC) is deleterious to cells [25]. Since ER has very low cholesterol content, the accumulation of FC in this cellular organelle may induce membrane dysfunction and subsequent ER stress. The majority of cholesterol in adipocytes originates from circulating lipoproteins, as a consequence of the low activity in cholesterol de novo synthetic pathway [26]. Previous study of Kuniyasu et al indicated that adipocytes may function as phagocytes like macrophages to uptake and degrade Ox-LDL [27].Our group also reported that adipocytes can endocytosis and degrade cholesterol from lipoproteins in the circulation [28–30]. In this study we found that cholesterol load in 3T3-L1-derived adipocytes increased with the ox-LDL incubation. Therefore it could be presumed that cholesterol load may be increased in adipocytes through endocytosis and degradation of ox-LDL, which subsequently result in the activation of ER stress. Though need further study, this provides a possible mechanism for the induction of adipocytes ER stress by ox-LDL.

Ox-LDL is a trigger of lipoprotein-associated oxidative stress. Oxidative stress is a common insult that can lead to ER stress. So ox-LDL may also induce ER stress through its oxidative stress properties. Marie Sanson et al. previously reported that treatment of human vascular endothelial and smooth muscle cells with ox-LDL induced a sustained rise of cytosolic calcium leading to the activation of ER stress. As we know, ox-LDL has multiple biological activities [14]. Other constituents and biological properties of ox-LDL may also lead to ER stress, which is worth further exploration.

In conclusion, this study demonstrated ox-LDL induced an increasing release of visfatin and resistin from 3T3-L1 adipocytes through activation of ER stress, suggesting ox-LDL may play a causative role in the development of secretion dysfunction in adipocytes. We also showed that adipocytes can take up ox-LDL in vitro leading to intracellular FC overload, which may be a possible mechanism for the induction of ER stress by ox-LDL. The present study provided a potential role of oxidated lipids in dysregulation of adipokines in obesity.
